# Effects of Endogenous Antioxidants in Camellia Oil on the Formation of 2-Monochloropropane-1, 3-diol Esters and 3-Monochloropropane-1,2-diol Esters during Thermal Processing

**DOI:** 10.3390/foods13020261

**Published:** 2024-01-14

**Authors:** Shanshan Liu, Mingyue Shen, Jianhua Xie, Bohan Liu, Chang Li

**Affiliations:** State Key Laboratory of Food Science and Resources, Nanchang University, Nanchang 330047, Chinashenmingyue1107@ncu.edu.cn (M.S.); jhxie@ncu.edu.cn (J.X.);

**Keywords:** MCPD esters, endogenous antioxidants, camellia oil, mechanism

## Abstract

2-Monochloropropane-1, 3-diol (2-MCPD) esters and 3-monochloropropane-1,2-diol (3-MCPD) esters, a class of substances potentially harmful to human health, are usually formed during the refining of vegetable oils under high temperature. The effects of endogenous antioxidants in vegetable oils on the formation of 2- and 3-MCPD esters is still unknown. In this study, the effects of endogenous antioxidants (α-tocopherol, stigmasterol and squalene) on the formation of 2- and 3-MCPD esters in model thermal processing of camellia oil were investigated. The possible formation mechanism of 2- and 3-MCPD esters was also studied through the monitoring of acyloxonium ions, the intermediate ions of 2- and 3-MCPD esters formation, and free radicals by employing infrared spectra and electron paramagnetic resonance (EPR), respectively. The results indicated that the addition of α-tocopherol had either promoting or inhibiting effects on the formation of 2- and 3-MCPD esters, depending on the amount added. Stigmasterol inhibited the formation of 3-MCPD ester and 2-MCPD ester at low concentrations, while promoting their formation at high concentrations. Squalene exhibited a promotional effect on the formation of 3-MCPD ester and 2-MCPD ester, with an increased promotion effect as the amount of squalene added increased. The EPR results suggested that CCl_3_•, Lipid alkoxyl, N_3_• and SO_3_• formed during the processing of camellia oil, which may further mediate the formation of chlorpropanol esters. This study also inferred that squalene promotes the participation of the free radical in chlorpropanol ester formation.

## 1. Introduction

Chloropropanol esters, widely found in fat-based food matrices and edible oils, have attracted increasing attention since they are a type of contaminant that possesses potential toxicity to human health [1,2]. It is reported that chloropropanol esters can be hydrolyzed by heat, acid, as well as by microbial and intestinal lipases to generate free chloropropanols [3]. According to the position and number of chloride substitution, chloropropanol esters mainly consist of 3-monochloropropane-1,2-diol (3-MCPD) esters, 2-monochloropropane-1,3-diol (2-MCPD) esters, 2,3-dichloropropane-ol (2,3-DCP) esters and 1,3-dichloropropane-2-ol (1,3-DCP) esters [4]. Among these substances, 2- and 3-MCPD esters are most abundant in refined vegetable oils. 3-MCPD has been verified as affecting male fertility and kidney function, and even inducing tumor in rats [5]. Currently, insufficient data are available draw conclusions on the toxicity of 2-MCPD esters. The International Agency for Research on Cancer (IARC) has characterized 3-MCPD as a possible carcinogen in humans (Group 2B) [6]. In response to the toxicity of 3-MCPD, some risk management programs have been introduced internationally. The Joint Food and Agriculture Organization/World Health Organization Expert Committee on Food Additives (JECFA) established a provisional maximum tolerable daily intake (PMTDI) of 4 µg kg^−1^ bw day^−1^ for 3-MCPD [7] and the European Union (EU) set a maximum limit of 0.02 μg g^−1^ for 3-MCPD in soy sauce. 

2-MCPD esters and 3-MCPD esters are widely present in refined vegetable oils. Palm oil was reported to have the highest concentrations of 2- and 3-MCPD esters compared to other oils [8,9]. MacMahon et al. [10] found that the content of 3-MCPD esters in unrefined oil was much lower than that in refined oil and the highest contents were detected in refined palm oils. Li and his co-workers came to a conclusion that 3-MCPD esters were generated with maximum values by 1–1.5 h at a certain deodorizing temperature (220–260 °C) in a study of formation of 3-MCPD esters in peanut oil during physical refining [11]. In addition, it was found that 3-MCPD esters increased with frying temperature in palm olein during the deep-fat frying of potato chips [2].

To the best of our knowledge, mitigation strategies for 3-MCPD esters in vegetable oil mainly include: optimization of the refining process, removal of precursor substances and the addition of antioxidants [12,13]. For instance, Sim et al. [14] optimized phosphoric acid dosage, degumming temperature, bleaching earth dosage and deodorization temperature in the physical refining of palm oil via the response surface methodology; the optimized processing conditions resulted in a more than 80% reduction in 3-MCPD esters. Some researchers have successfully used trapping agents to deplete precursor organochlorines from sunflower and soybean oils, the levels of MCPD esters decreased 60–80% [15]. Natural and synthetic antioxidants have been added in palm olein when frying potato chips, 3-MCPD esters in both fried potato chips and oils were mitigated to some extent [16]. Many antioxidants such as tocopherol, phytosterol and squalene widely exist in various vegetable oils [17], such as camellia oil. Camellia oil is an edible oil from *Camellia oleifera* Abel species of the camellia family, which is widely cultivated in the south of China [18]. Camellia oil contains various natural antioxidants; however, previous investigations have shown that it contains a high content of 3-MCPD esters [19,20]. Up to now, there are few studies on the effect of endogenous antioxidant components on the formation of MCPD esters in vegetable oils. 

The objective of this study was to investigate the impact of α-tocopherol, stigmasterol and squalene on the formation of 2- and 3-MCPD esters in camellia oil during heating and to investigate the possible formation mechanism of 2- and 3-MCPD esters.

## 2. Materials and Methods

### 2.1. Materials and Chemicals 

Analytical standards: 3-monochloropropane-1,2-diol (3-MCPD) ester, 2-monochloropropane-1,2-diol (2-MCPD) ester, d_5_-3-MCPD-1,2-bis-palmitoyl (d_5_-3-MCPD) ester and d_5_-2-MCPD-1,3-bis-palmitoyl (d_5_-2-MCPD) ester were procured from Toronto Research Chemicals Inc. (North York, Toronto, ON, Canada). Squalene and α-tocopherol were purchased from Aladdin Bio-chemical Technology Co., Ltd. (Shanghai, China), stigmasterol was obtained from Sigma-Aldrich (St. Louis, MO, USA). 1-monopalmitin and 1,2-palmitate diester were purchased from Shanghai Yiji Industrial Co., Ltd. (Shanghai, China). 5,5-Dimethyl-1-pyrroline N-oxide (DMPO) and N-tert-Butyl-α-phenylnitrone (PBN) were supplied by Merck Company (Darmstadt, Germany). Tert-butyl methyl ether, isohexane, isooctane and ethyl acetate were of chromatographic pure grade. Phenylboronic acid (PBA), anhydrous sodium sulphate, sodium bromide, anhydrous sodium sulfate, sodium methoxide, sodium chloride, iron trichloride, sulphuric acid, diethyl ether and methylbenzene were of analytical grade. Crude camellia oil was supplied by a local manufacturer and silica gel was obtained from Aladdin bio-chemical technology Co., Ltd. (Shanghai, China). 

### 2.2. Methods 

#### 2.2.1. Oil Sample Preparation 

Crude camellia oil was degummed by adding phosphoric acid (0.15%, *w*/*w*) and water (5%, *w*/*w*), and heated at 85 °C for 30 min with continuous stirring. After centrifuging at 4500 rpm for 5 min, the degummed oil was obtained from the upper layer. Degummed oil was eluted on a silica gel column by a mixture of ethyl acetate and petroleum ether (6:4, *v*/*v*). The eluent was collected and the solvent was evaporated by a rotary evaporator to acquire an oil sample. 

#### 2.2.2. Effects of Heating Temperature on the Formation of 2- and 3-MCPD Esters in the Model System

Two grams of oil sample prepared according to Section 2.2.1 was mixed with 100 μL 5.1 mol L^−1^ NaCl (or 0.1 mol L^−1^ FeCl_3_) in a thick-walled pressure-resistant glass tube. The mixtures were heated at 240, 260, 280 and 300 °C, respectively, for 1.0 h in a sand bath, then cooled to ambient temperature.

#### 2.2.3. Effects of Heating Time on the Formation of 2- and 3-MCPD Esters in the Model System

Two grams of oil sample prepared according to Section 2.2.1 was mixed with 100 μL 5.1 mol L^−1^ NaCl (or 0.1 mol L^−1^ FeCl_3_) in a thick-walled pressure-resistant glass tube. The mixtures were heated at 280 °C for 0, 0.5, 1.0 and 2.0 h in a sand bath, respectively, then cooled to ambient temperature. 

#### 2.2.4. Influence of Three Endogenous Antioxidants on the Formation of 2- and 3-MCPD Esters in the Model System 

Two grams of oil sample prepared according to Section 2.2.1 and 100 μL 0.1 mol L^−1^ FeCl_3_ were mixed in a thick-walled pressure-resistant glass tube, then 20 μL of antioxidants (α-tocopherol, stigmasterol and squalene) with different concentrations were added, respectively. The mixtures were heated at 280 °C for 1.0 h in a sand bath. Finally, the mixtures were cooled to ambient temperature.

#### 2.2.5. Determination of 2- and 3-MCPD Esters by GC-MS

An oil sample (100 ± 0.5 mg) was weighed into a 5 mL tube, and then 10 μL of d_5_-3-MCPD and d_5_-2-MCPD esters were added. The mixture was completely dissolved in 100 μL of tert-butyl methyl ether. After mixing, 200 μL of sodium methoxide solution was added to make an ester cleavage for 5 min, then the reaction was terminated with 600 mL of acidified sodium bromide. Afterwards, 600 mL of iso-hexane was added to remove the upper layer of the organic phase. The aqueous phase was extracted 3 times with 600 μL of diethyl ether and ethyl acetate (3:2, *v*/*v*) and was combined in a new tube containing 2 mg of anhydrous sodium sulphate, followed by the addition of 100 μL of the derivatizing reagent (PBA). The reaction was proceeded at ambient temperature for 30 min and dried under nitrogen. Finally, the residue was dissolved in 1.0 mL of chromatographic grade isooctane and filtered through a 0.22 μm nylon membrane. 

The analysis of 2- and 3-MCPD esters was carried out using an Agilent 7890B GC system equipped with a 7000D Triple Quadrupole mass spectrometer (Agilent Technologies Inc., Santa Clara, CA, USA). A HP-5 MS capillary column (60 m × 0.25 mm, 0.25 μm) was used for separation. The carrier gas was helium with a constant flow rate of 1.0 mL min^−1^. The oven temperature was programmed at: 85 °C (0.5 min), from 85 °C to 180 °C at 12 °C min^−1^, from 180 °C to 280 °C at 40 °C min^−1^, isothermal 7.16 min. The data was acquired by using the selected ion monitoring (SIM) mode. The quantitative analysis was carried out by monitoring the characteristic ions (quantifier) at m/z147 (3-MCPD), m/z196 (2-MCPD), m/z150 (d_5_-3-MCPD) and m/z201 (d_5_-2-MCPD). The ions at m/z196 (3-MCPD), m/z198 (2-MCPD), m/z201 (d_5_-3-MCPD) and m/z203 (d_5_-2-MCPD) were used as qualifiers.

#### 2.2.6. Monitoring of Acyloxonium Ions in the Model System

Four aliquots of oil samples were placed in 4 separate thick-walled pressure-resistant glass tubes, respectively, then were heated at 240 °C for 5 min, 10 min, 20 min and 30 min, respectively, then were cooled to ambient temperature. Another 4 aliquots of oil samples with 200 μL 0.1 M FeCl_3_ were mixed in 4 separate thick-walled pressure-resistant glass tubes, respectively; the mixtures were heated at 240 °C for 5 min, 10 min, 20 min and 30 min in a sand bath, then were cooled to ambient temperature. Unheated samples were used as controls. Acyloxonium ions were monitored by Fourier infrared spectrometer with a deuterated triglyceride sulfate (DTGS) detector and an OMNI sampler. The scan mode was attenuated total reflection (ATR), the spectral range was 650–4000 cm^−1^, the scan frequency was 4 cm^−1^, and the cumulative scan was 64 times. 

#### 2.2.7. Free Radical Analysis in the Model System

The oil samples were weighed into thick-walled pressure-resistant glass tubes, and α-tocopherol, stigmasterol and squalene were added to each sample, respectively. PBN was used as spin trapping reagent. The mixture was heated at 220 °C for 20 min in a sand bath, then transferred into a capillary glass tube to detect spin signals in ESR. The EPR central magnetic field was set at 3420 G, the scanning width was 200 G, the scanning time was 10 s, the receiving gain was 40 dB, the scanning was performed 30 times, and the microwave power was 25 dB. 

After scanning according to the set parameters, the free radical absorption curve area was selected, and the differential signal was integrated twice to calculate the number of free radicals.

#### 2.2.8. Statistical Analysis 

All data were acquired by implementing the experiment in triplicate and expressed as the mean value ± standard deviation. The experiment data were analyzed by Origin Pro (version 94E) software (Stat-Ease Inc., Minneapolis, MN, USA).

## 3. Results and Discussion

### 3.1. Effects of Heating Temperature on the Formation of 2- and 3-MCPD Esters

**Figure 1:**
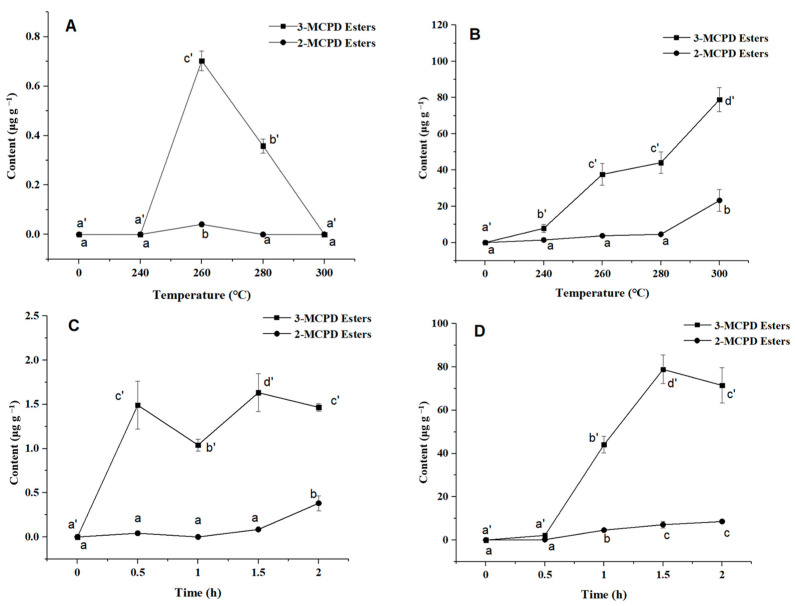
Influence of heating temperature and time on the formation of 3-MCPD and 2-MCPD esters in the NaCl (**A**,**C**) and FeCl_3_ (**B**,**D**) systems. Different lowercase letters indicate a significant difference in the level of 2-MCPD esters (*p* < 0.05), while different lowercase letters with an apostrophe indicate a significant difference in the level of 3-MCPD esters (*p* < 0.05).

2- and 3-MCPD esters formed at different heating temperatures are shown in Figure 1. In the NaCl system (Figure 1A), when it was heated to 260 °C or 280 °C, a small number of 3-MCPD esters was generated, while when it was heated to 240 °C or 300 °C, 3-MCPD esters were not detected in the system. It is suggested that 3-MCPD esters decomposed with the rising temperature and that the decomposition rate was greater than the formation rate. However, 2-MCPD esters were hardly generated under any of the heating temperatures (Figure 1A). The amounts of 3-MCPD esters in the FeCl_3_ system (Figure 1B) significantly increased with the rise in heating temperature, with the maximum rising rates observed between 240 °C and 260 °C and between 280 °C and 300 °C. 2-MCPD esters rose gently from 240 °C to 280 °C and increased sharply at 300 °C (Figure 1B). 

### 3.2. Effects of Heating Time on the Formation of 2- and 3-MCPD Esters 

The oil samples were heated at 280 °C for different times. The effects of heating time on the formation of 2- and 3-MCPD esters are shown in Figure 1 C-D. In the NaCl system (Figure 1C), the content of 3-MCPD esters increased rapidly when heated from 0 h to 0.5 h, then presented a dynamic equilibrium state, indicating that the 3-MCPD ester was constantly formed during the heating process and decomposed under high temperature simultaneously. The contents of the 2-MCPD ester were not significantly different at 0 h, 0.5 h, 1 h and 1.5 h (*p* < 0.05), and reached a maximum at 2 h. In the FeCl_3_ system (Figure 1D), the amounts of 3-MCPD esters exhibited a trend first of a sharp increase (0.5–1.5 h) and then obvious decreasing (1.5–2 h), yet with no significant difference between 0 h and 0.5 h (*p* < 0.05), while 2-MCPD ester contents increased slowly and steadily. 

From Figure 1, the contents of 3-MCPD ester were higher than 2-MCPD ester under different heating temperatures and times, which was consistent with previous studies [21,22]. The reason for this result is that the sn-1,3 locus is more susceptible to attack than the sn-2 locus, making it easier to form 3-MCPD ester. The contents of 2- and 3-MCPD esters were much higher and their formation was more stable in the FeCl_3_ system compared to the NaCl system, which is consistent with the findings of a previous study [23]. The FeCl_3_ system not only provides the necessary chlorine atoms for chlorpropanol ester formation but also acts as a catalytic agent, Fe^3+^, for this reaction. Additionally, the Fe^3+^ system promotes the generation of free radicals and forms more stable complex substances by combining with free radical intermediates due to its unique electronic structure [24]. 

### 3.3. Effects of Endogenous Antioxidants on the Formation of 3-MCPD and 2-MCPD Esters

**Figure 2:**
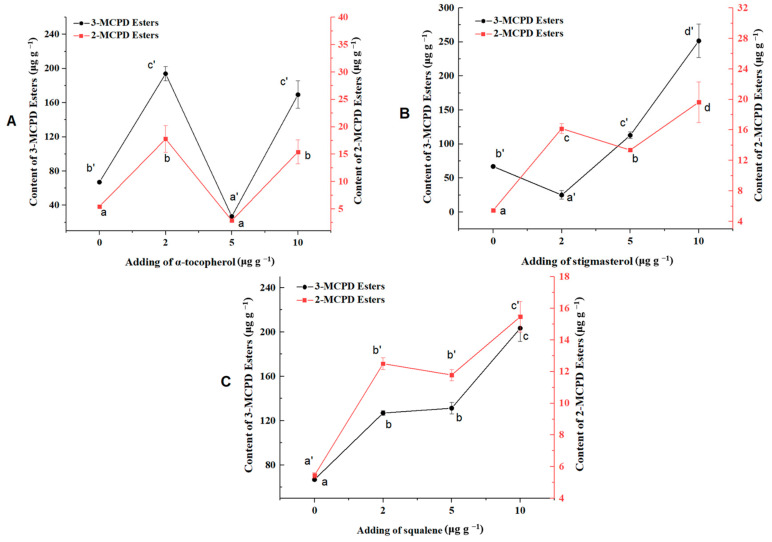
Influence of endogenous antioxidants ((**A**), α-tocopherol; (**B**), stigmasterol; (**C**), squalene) on the formation of 2 and 3-MCPD esters in the FeCl_3_ system. Different lowercase letters indicate a significant difference in the level of 3-MCPD esters (*p* < 0.05), while different lowercase letters with an apostrophe indicate a significant difference in the level of 2-MCPD esters (*p* < 0.05).

Stigmasterol, α-tocopherol and squalene were commonly found in camellia oil [25]. In this study, the effects of these three compounds on the formation of 2- and 3-MCPD esters were investigated. The formation of 3-MCPD esters and 2-MCPD esters were promoted when the amount of α-tocopherol added was 2 μg g^−1^ or 10 μg g^−1^, compared with the control, while 3-MCPD esters were inhibited when the amount of α-tocopherol added was 5 μg g^−1^ (Figure 2A). When stigmasterol was added to the system, compared to the blank control, the content of 3-MCPD ester initially decreased and then increased as the additions increased. On the other hand, 2-MCPD ester exhibited a trend of sharp increase followed by decrease, and then another increase (Figure 2B). The contents of 2- and 3-MCPD esters increased with the increasing amounts of squalene and were higher than those in the blank control (Figure 2C), suggesting that the presence of squalene promoted the formation of 3-MCPD and 2-MCPD esters. 

### 3.4. Monitoring of Acyloxonium Ions 

**Figure 3:**
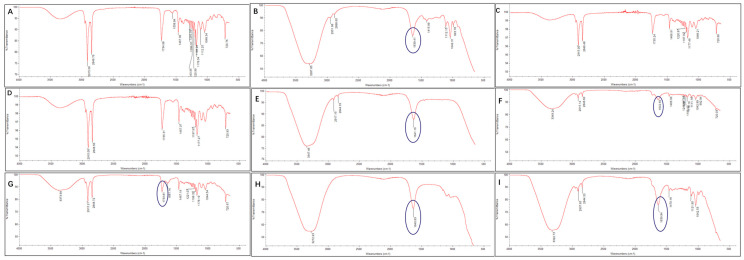
Infrared spectra of oil (**A**–**E**) and oil + FeCl_3_ (**F**–**I**) heated at 240 °C for different times. (**A**): Control, (**B**): 5 min, (**C**): 10 min, (**D**): 20 min, (**E**): 30 min; (**F**): 5 min, (**G**): 10 min, (**H**): 20 min, (**I**): 30 min.

Acyloxonium ions are the intermediates associated with the formation of chlorpropanol esters. They exhibit a characteristic absorption peak at 1651 cm^−1^ during infrared spectral scanning [26]. This study employed Fourier transform infrared spectroscopy (FT-IR) to monitor acyloxonium ions. As shown in Figure 3, the measurement results in this study have an overall offset of 10 wavenumbers to the right. Since 1641 cm^−1^ falls within the range of the characteristic absorption peak of acyloxonium ions, which is considered as the characteristic absorption peak of acyloxonium ions. When the oil sample was not heated, no characteristic peak of acyloxonium ions was observed (Figure 3A). However, after heating for 5 min, significant changes in the infrared absorption peaks were observed and the peak corresponding to acyloxoniums ion appeared (Figure 3B). The absorption peak at 1641 cm^–1^ disappeared after being heated for 10 and 20 min (Figure 3C,D). The characteristic absorption peak of acyloxonium ions reappeared when it was heated for 30 min (Figure 3E). This indicated that the unstable acyloxonium ions were formed during heating, as acyloxonium ions acted as an activated complex [24]. It can be observed in Figure 3A–E that when the absorption peak at 1641 cm^−1^ appears, the absorption peak of the carboxyl group at 1735 cm^−1^ significantly tends to disappear, indicating that the carboxyl group participated in the reaction during heating. 

Chloride ions are essential to the formation of chlorpropanol esters. Oil was heated with FeCl_3_ for infrared measurement in this study, and the results are shown in Figure 3F–I. When heated for 5 and 10 min, a weak absorption peak appeared at 1641 cm^−1^, suggesting the formation of a small amount of acyloxonium ions in the system. Subsequently, by continuing to heat for 20 and 30 min, the absorption peak of acyloxonium ions was significantly enhanced. As the acyloxonium ions gradually formed, the absorption peak at 1733 cm^−1^ of the carboxyl groups also disappeared, which was consistent with previous results [23,24]. 

### 3.5. Monitoring Free Radicals

Some researchers have proposed a free radical mechanism for the formation of chloropropanol esters [23,24]. For example, Zhang et al. demonstrated that iron ions promote free radical formation, and then catalyze the formation of 3-MCPD esters [24]. In the present study, electron spin resonance was used to monitor the free radicals generated in camellia oil during heating. 

#### 3.5.1. Selection of the Spin-Trapping Reagent

**Figure 4:**
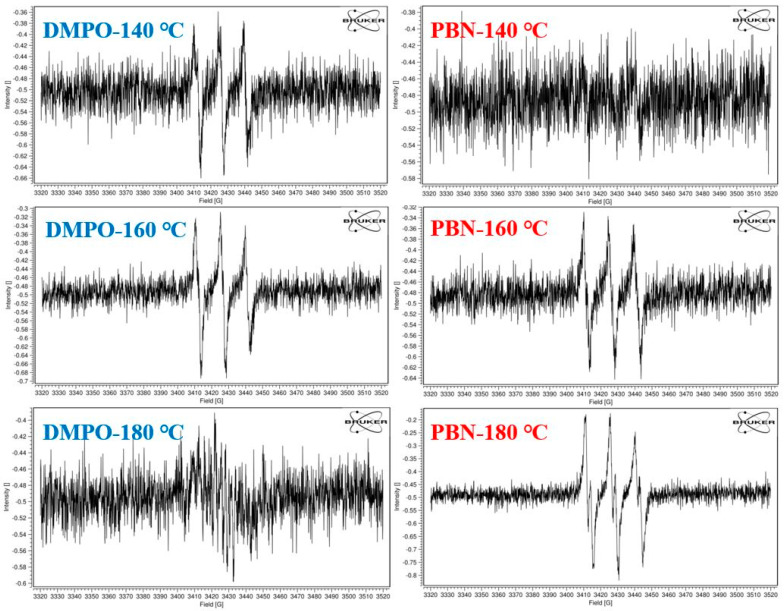
Spectra of free radical signals in camellia oil at different heating temperatures, using DMPO or PBN as the trapping reagent.

The most commonly used technique for studying radicals is electron spin resonance (ESR)/electron paramagnetic resonance (EPR) spectroscopy. However, the high reactivity of free radicals causes them to have extremely short lifetimes, which poses difficulties for studying radicals. The most direct way to extend the life of radicals is adding a trapping agent that can form more stable radical adducts with the original free radicals [27]. Both PBN and DMPO have been widely used to trap radicals in previous studies [28,29]. In this study, PBN and DMPO were tested as spin-trapping reagents to determine their appropriateness. Figure 4 shows the spectra of free radical signals when camellia oil was heated at different temperatures. When DMPO was used as a trapping reagent, the free radical signal was stronger at 160 °C than at 140 °C, but almost disappeared when the temperature increased to 180 °C. This may be due to DMPO’s poor thermal stability, which causes it to decompose readily at higher temperatures. Compared with DMPO, PBN is more stable at higher temperatures and was therefore chosen as the trapping reagent in subsequent experiments.

#### 3.5.2. Identification of Free Radicals in Camellia Oil

**Figure 5:**
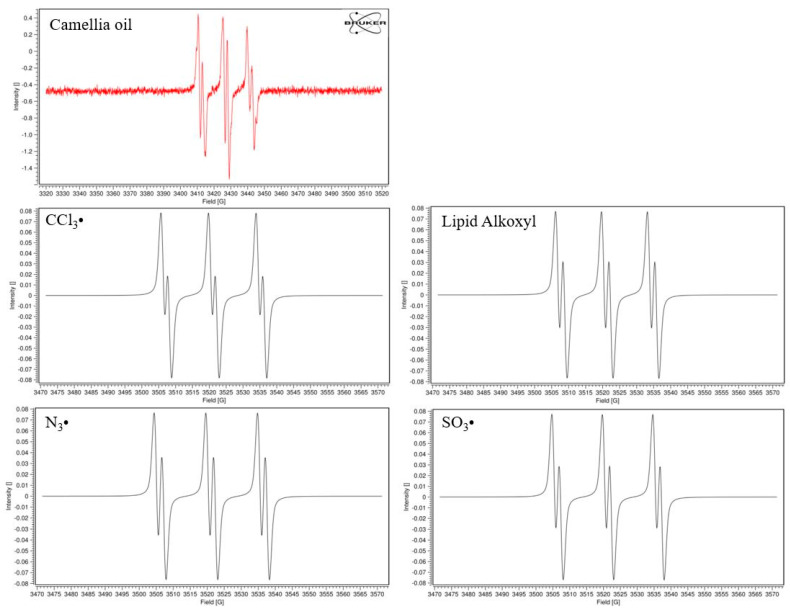
The free radical signals of CCl_3_•, Lipid alkoxyl, N_3_• and SO_3_• and radicals formed in camellia oil captured by PBN.

In this study, the free radical signal detected in camellia oil was matched with the signals in the equipped library, the most likely free radical species (CCl_3_•, lipid alkoxyl, N_3_• and SO_3_•) were listed in Figure 5. Therefore, this study speculated that CCl_3_•, lipid alkoxyl, N_3_• and SO_3_• free radicals formed in camellia oil during thermal processing.

#### 3.5.3. Effects of Endogenous Antioxidants on the Formation of Free Radicals 

**Figure 6:**
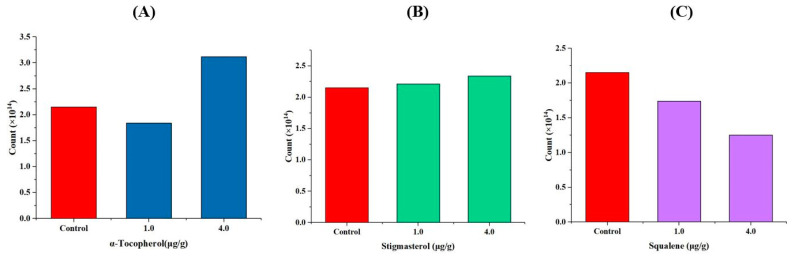
The free radical count in camellia oil after adding different amounts of α-tocopherol (**A**), stigmasterol (**B**) and squalene (**C**).

In order to investigate the effects of endogenous antioxidants on the formation of free radicals in camellia oil, different amounts (1 μg g^−1^ and 4 μg g^−1^) of α-tocopherol, stigmasterol and squalene were added to camellia oil before being heated. Quantification of the corresponding free radicals is shown in Figure 6. Compared with the blank, addition of α-tocopherol with a concentration of 1 μg g^−1^ in camellia oil could lower the number of free radicals, while addition with 4 μg g^−1^ could increase the number of free radicals (Figure 6A). Adding 1 μg g^−1^ and 4 μg g^−1^ of stigmasterol had no significant effect on the strength of the radical signal in the camellia oil compared to the blank control (Figure 6B). While the free radical amount in the camellia oil was obviously reduced when squalene was added, it was lower at 4 μg g^−1^ than at 1 μg g^−1^ (Figure 6C). All in all, the addition of α-tocopherol, stigmasterol and squalene expressed no effect on the type of free radicals, only affecting the strength of the radical signal. Jerzykiewicz et al. studied the oxidation of fatty acids and triglycerides with α-tocopherol by the radicals generated from H_2_O_2_ and found that this process is inhibited by lower concentrations of α-tocopherol, whereas it is accelerated by higher concentrations of α-tocopherol [30]. Zuta et al. also found that higher concentrations of α-tocopherol were less effective in controlling oxidation in the oils than lower α-tocopherol levels [31]. This suggested that the high concentration of alpha tocopherol may not inhibit the formation of free radicals under certain conditions; instead, it may potentially facilitate this process. In terms of stigmasterol, as a phytosterol, its antioxidant activity is still inconclusive. For instance, Gordon et al. believed that stigmasterol had no significant antioxidant activity in edible oils [32]. This may explain the result obtained in our study. While Yoshida et al. found that stigmasterol accelerated the oxidation of both methyl linoleate in solution and β-linoleoyl-γ-palmitoyl phosphatidylcholine liposomal membranes in aqueous dispersions [33]. This difference may be attributed to the solvent environment in which stigmasterol is situated. Similarly, the antioxidant activity of squalene is also controversial. Squalene, containing multiple unsaturated double bonds, is prone to oxidation, which helps prevent the oil from oxidizing. Psomiadou et al. found that the role of squalene in olive oil stability in terms of antioxidant activity is not significant, and they considered that the weak antioxidant activity of squalene in olive oil may be explained by competitive oxidation of the different lipids present, which leads to a reduction of the oxidation rate [34]. 

## 4. Conclusions

This study investigated the effects of endogenous antioxidants on the formation of 2- and 3-MCPD esters in a model thermal processing of camellia oil, and the possible formation mechanism of 2- and 3-MCPD esters through the monitoring of acyloxonium ions and free radicals. The antioxidants presented both prompting and inhibiting effects on the formation of 2- and 3-MCPD esters; in particular, squalene showed a clear promoting effect on the formation of 2- and 3-MCPD esters. Acyloxonium ions were monitored in our study, which also confirmed that they are an intermediate ion for chloropropanol ester formation. Additionally, coupled with the detection results of free radicals, we hypothesized that these effects are due to complex chemical reaction occurred between antioxidants and free radicals, such as the CCl_3_•, Lipid alkoxyl, N_3_• and SO_3_• formed during thermal processing, which further promote the formation of 2- and 3-MCPD esters. In conclusion, the endogenous antioxidants in camellia oil have a significant impact on the formation of MCPD esters, and this specific effect is related to the type and content of antioxidant substances. The shortcoming of this study is that only three antioxidants in camellia oil were selected, and future studies will select more endogenous antioxidants for investigation to acquire additional data and provide evidence uncovering the reasons behind the high MCPD ester content in camellia oil. This study could provide some references for the relevant research of chloropropanol esters in vegetable oils.

## Data Availability

The data presented in this study are available on request from the corresponding author. The data are not publicly available due to privacy restrictions.
